# SARIMA and ARDL models for predicting leptospirosis in Anuradhapura district Sri Lanka

**DOI:** 10.1371/journal.pone.0275447

**Published:** 2022-10-13

**Authors:** Janith Warnasekara, Suneth Agampodi, Abeynayake NR

**Affiliations:** 1 Department of Community Medicine, Faculty of Medicine and Allied Sciences, Rajarata University of Sri Lanka, Mihintale, Sri Lanka; 2 Postgraduate Institute of Agriculture, University of Peradeniya, Peradeniya, Sri Lanka; 3 Department of Agribusiness Management, Faculty of Agriculture and Plantation Management, Wayamba University of Sri Lanka, Kuliyapitiya, Sri Lanka; Faculty of Medical Sciences, University of Sri Jayewardenepura, Sri Lanka, SRI LANKA

## Abstract

Leptospirosis is considered a neglected tropical disease despite its considerable mortality and morbidity. Lack of prediction remains a major reason for underestimating the disease. Although many models have been developed, most of them focused on the districts situated in the wet zone due to higher case numbers in that region. However, leptospirosis remains a major disease even in the dry zone of Sri Lanka. The objective of this study is to develop a time series model to predict leptospirosis in the Anuradhapura district situated in the dry zone of Sri Lanka. Time series data on monthly leptospirosis incidences from January 2008 to December 2018 and monthly rainfall, rainy days, temperature, and relative humidity were considered in model fitting. The first 72 months (55%) were used to fit the model, and the subsequent 60 months(45%) were used to validate the model. The log-transformed dependent variable was employed for fitting the Univariate seasonal ARIMA model. Based on the stationarity of the mean of the five variables, the ARDL model was selected as the multivariate time series technique. Residuals analysis was performed on normality, heteroskedasticity, and serial correlation to validate the model. The lowest AIC and MAPE were used to select the best model. Univariate models could not be fitted without adjusting the outliers. Adjusting seasonal outliers yielded better results than the models without adjustments. Best fitted Univariate model was ARIMA(1,0,0)(0,1,1)_12_,(AIC-1.08, MAPE-19.8). Best fitted ARDL model was ARDL(1, 3, 2, 1, 0),(AIC-2.04,MAPE-30.4). The number of patients reported in the previous month, rainfall, rainy days, and temperature showed a positive association, while relative humidity was negatively associated with leptospirosis. Multivariate models fitted better than univariate models for the original data. Best-fitted models indicate the necessity of including other explanatory variables such as patient, host, and epidemiological factors to yield better results.

## Introduction

Leptospirosis is a main tropical disease affecting humans as well as animals [1]. It is caused by a spirochete (a type of spiral-shaped bacteria) of the genus *Leptospira* of the family Leptospiraceae [2]. *Leptospira* is excreted to the environment via the urine of infected or carrier animals and enters the human body through a skin breach or a mucous membrane [2]. The severity of leptospirosis varies from mild self-resolving fever to severe disease with the involvement of major organs of the body [3]. These severe cases are considered in disease burden estimations, while mild cases are often neglected from these estimations in most settings [4]. The annual global burden of leptospirosis includes nearly one million cases, 60,000 deaths, and 2.9 million disability-adjusted life years [3]. However, these estimates are considered underestimations due to various reasons, including under notification, deficiencies of diagnostics, diversity of *Leptospira*, and inaccurate predictions [5–7]. Among the reasons for underestimation mentioned above, the major focus of this paper is to describe the gaps associated with disease predictions and propose prediction models for the Anuradhapura district of Sri Lanka.

Global disease estimations named Sri Lanka a hotspot of leptospirosis [8, 9]. The estimated annual incidence of leptospirosis is 52.2 per 100,000, with estimated annual deaths of 760 [4]. Nevertheless, these cases and deaths have not been distributed evenly across all parts of the country [4, 10]. Sri Lanka has three main climate zones: wet, dry, and intermediate zones [5]. The wet zone receives higher average annual rainfall than the other two zones and has higher humidity. At the same time, solar radiation and temperature are higher in the dry zone [5]. Leptospirosis outbreaks are usually associated with events due to heavy rainfalls such as floods or water-related recreational activities [11, 12]. Also, leptospirosis shows associations with the other meteorological parameters such as rainy days per month, solar radiation, relative humidity, and environmental temperature [5, 13–16]. Since both meteorological parameters and leptospirosis cases are changing across different geographical locations, the association of leptospirosis with meteorological parameters could be different in different regions [5].

**Fig 1:**
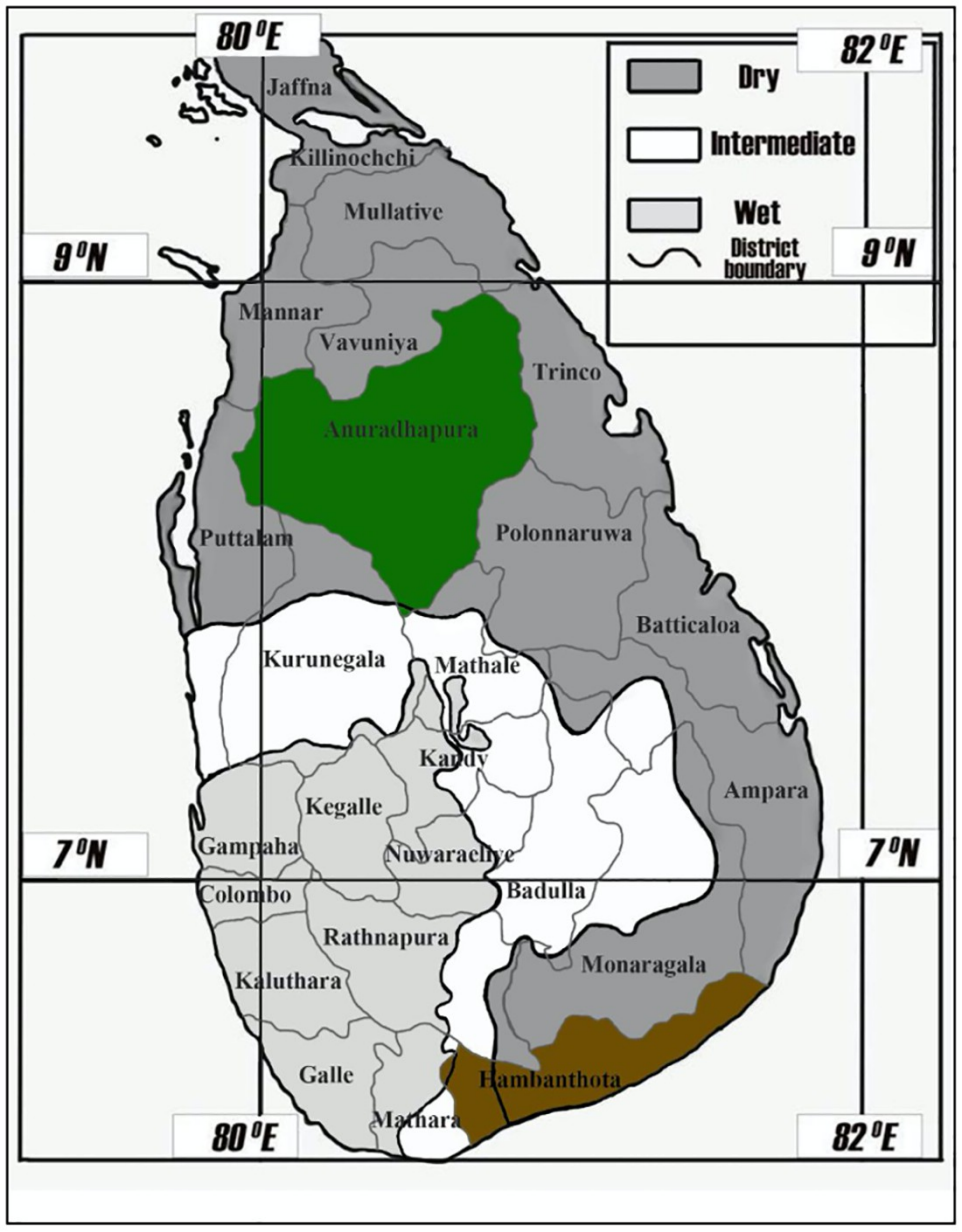
Three climate zones and the districts of Sri Lanka. Green: study setting; Anuradhapura district; Brown: Hambanthota district.

Many prediction models have been created to predict leptospirosis in Sri Lanka [5, 17, 18]. However, almost all studies have focused on a single or few districts for the modeling. Focusing on a single district may not represent the other districts, especially the ones situated in other climate zones. Also, most of the models developed were univariate, while multivariate techniques have not been adequately used for modeling in Sri Lanka [13]. further, only a few papers have targeted the dry zone of Sri Lanka for the prediction models [5, 18]. A previous study has demonstrated the differences between the climate zones regarding the prediction models and associations with meteorological parameters of leptospirosis [5]. It has been shown that the dry zone behaves entirely different from the wet zone of Sri Lanka with regard to patient reporting, outbreaks, rainfall, and other meteorological parameters [5]. However, in that study, the authors have not demonstrated the leptospirosis reporting pattern of the individual districts. Demonstrating the models for individual districts is essential as the dry zone extends from the northern part to the southern part of Sri Lanka. The only paper targeting an individual district in the dry zone was Ehelepola et al., published in 2021 [19]. Here, they have demonstrated that the temperature shows an opposite effect in the Kandy district (wet zone) in comparison to the Hambanthota district (dry zone). However, as shown in Fig 1, Hambanthota is not only situated in the dry zone. Therefore, selecting a district situated exclusively in the dry zone is essential to examine the meteorological association of leptospirosis in the dry zone where many predisposing factors for leptospirosis, such as paddy farming, are located.

We have selected the Anuradhapura district for this study to represent only the dry zone of Sri Lanka. Also, the Anuradhapura district has reported leptospirosis incidence of more than 500 per 100,000 population during the recent past [20]. Based on the ongoing research activities, the awareness, diagnosis, and reporting are expected to be high. Unlike in the districts of the wet zone, dry zone districts report episodic leptospirosis cases. Frequent wetness in wet zone facilitates *Leptospira* causing frequent outbreaks. In contrast, leptospirosis outbreaks in the Anuradhapura district are associated with northeastern monsoon rains from November to January [5]. Anuradhapura reports one of the highest incidents of leptospirosis among the districts in the dry zone [10]. Also, several micro geographical differences related to leptospirosis were noted in the Anuradhapura district compared to the Kandy district of Sri Lanka [12]. In addition, different types of *Leptospira* have been isolated from the dry zone compared to the wet zone [21]. Therefore, the Anuradhapura district is an ideal geographical setting to study the variations of leptospirosis and also to determine meteorological associations of leptospirosis in the dry zone of Sri Lanka. This study aims to develop mathematical models to predict leptospirosis and determine meteorological associations of leptospirosis in the Anuradhapura district of Sri Lanka.

## Methodology

### Study setting

The setting of this study was the Anuradhapura district of Sri Lanka. (Fig 1) It is situated in the dry zone and is considered a geographical area with typical features of the dry zone. Annual rainfall is less than 1750mm. The main rainy season is from November to January and is associated with northeastern monsoon rains [5, 22]. During this period, a higher number of leptospirosis cases are being reported. The majority of the population are paddy farmers who engage in paddy cultivation during the monsoon rains [23]. Most of the common socio-demographic risk factors are prevalent in this area [24]. Many papers have been published related to leptospirosis in Anuradhapura, Sri Lanka; nevertheless, none of the studies focused on time series analysis to forecast the future incidence of the district [12, 25–27].

### Data sources

Leptospirosis data are publicly available on the official webpage of the Epidemiology Unit of Sri Lanka [28]. Therefore, all the data published in the weekly epidemiological report were compiled as monthly data. In addition, data on monthly rainfall, rainy days, relative humidity, and environmental temperature were purchased from the meteorological department of Sri Lanka [22]. The period for the obtained data was from January 2008 to December 2018.

### Data analysis

**Fig 2:**
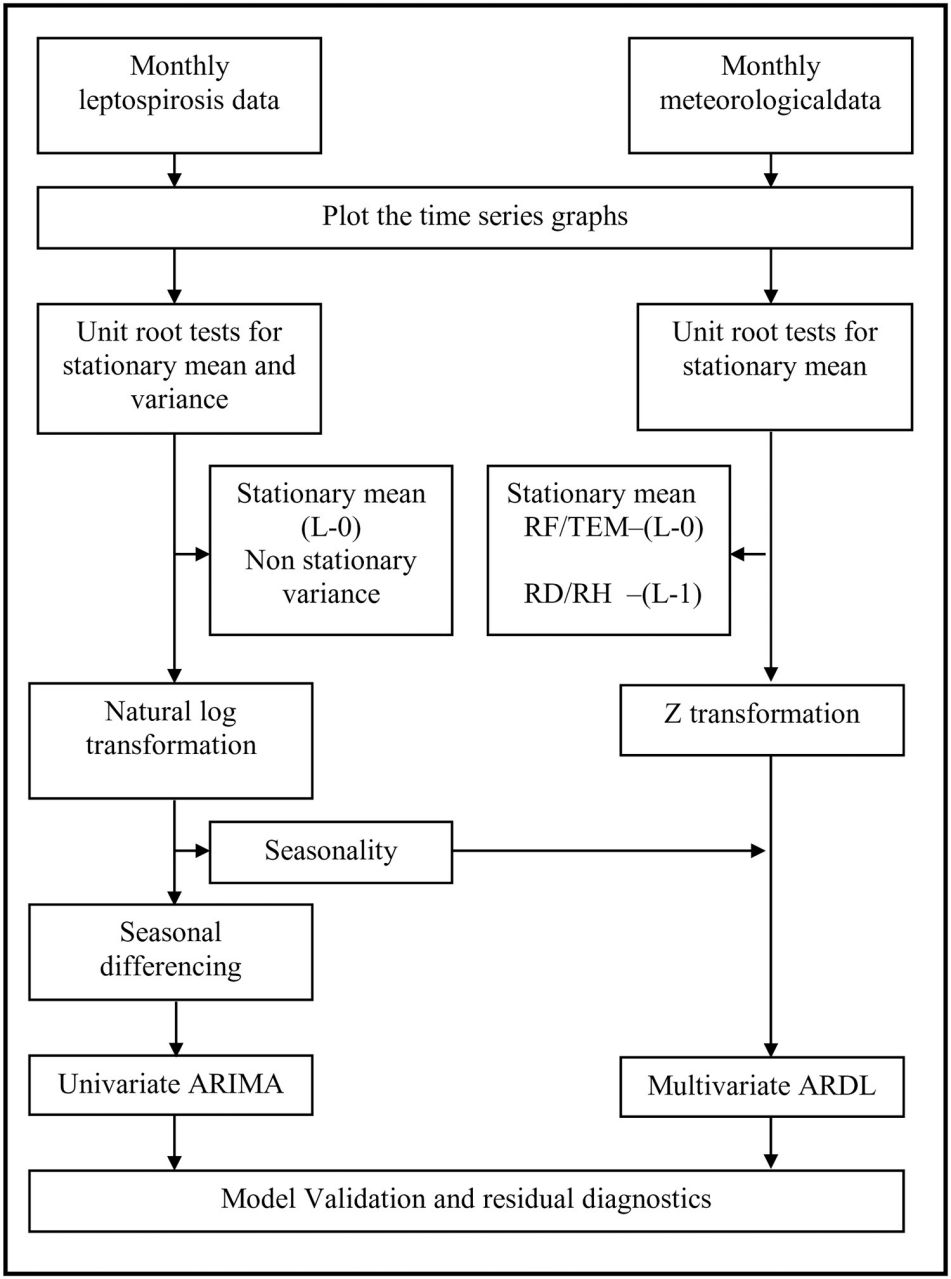
Flow chart of the data analysis and model fitting. RF-rainfall, TEM-temperature, RD-rainy days, RH-relative humidity.

The data analysis was done using Eviews version 10. Fig 2 summarises the analytical framework of data analysis.

### Testing for stationary and seasonality

The stationary mean was confirmed using the unit root test by the Augmented Dickey-Fuller test. (Table 1) In the unit root test, the stylized trend-cycle decomposition of a time series y_t_ is given by

$$y_{t}={TD}_{t}+z_{t}$$

where

TD_t_ is a deterministic linear trend, and z_t_ is the AR(1) process.

**Table 1:** Standard and seasonal unit root test results of the variable of the natural log-transformed monthly leptospirosis cases.

	Augmented Dickey-Fuller test for stationary mean	Traditional HEGY process for stationary seasons
Frequency	Non-seasonal	12 months per cycle
1% significance level	-2.88	33.9
5% significance level	-3.48	8.15
Test statistic	-6.41	9.88
Interpretation	The mean is stationary at a 1% significance	Seasonality is significant at 1% significance

The seasonality of the data series was tested using the traditional Hylleberg, Engle, Granger, and Yoo(HEGY) process. (Table 1).

### Data transformation and differencing

The monthly leptospirosis incidence distribution had non-stationary variance and stationery mean while showing a significant seasonality effect. Natural log transformation was done to achieve stationary variance. The original value of ‘0’ is identified as a missing value during the log transformation, and the value ‘1’ becomes 0. Linear interpolation using Eviews was performed to avoid the effect on original values of 0 and 1 during log transformation. Regular differencing was not performed as the mean was stationary. Instead, seasonal differencing was performed to remove the seasonality of data. In multivariate analysis, all regressors were Z transformed to avoid the measuring unit effect of the variables.

### Outlier adjustment

Outlier adjustment was performed only for univariate analysis as residuals did not follow normal distribution without adjustment for seasonal outliers. Z scores were obtained for the seasonality-adjusted data. Z scores of more than 1.96 were adjusted using the linear interpolating function of Eviews. In the linear interpolation method, linear approximation was made based on the previous and the next non-missing value of the series as an equation and the interpolated value(Iv) is given by,

$$Iv=\left(1-\lambda \right)P_{i-1}+\lambda P_{i+1}$$

where

λ is the relative position of the missing value, P_(i-1)_is the immediately previous value, and P_(i+1)_ is the immediate next missing value.

### Model fitting

The first 72 time points (January 2008 to December 2013) were used to create the models, and the following 60-time points(January 2014 to December 2018) were used to validate them in both univariate and multivariate modeling.

### Univariate analysis

The autocorrelation function (ACF) and Partial autocorrelation function (PACF) of the variable of monthly leptospirosis patients were plotted. Based on the significant lags of ACF and PACF, the Seasonal ARIMA model was fitted. The model designation ARIMA (p, d, q) (P, D, Q)_s_ consists of regular autoregressive (AR-p) and moving average (MA-q) terms, which account for correlations with low lags. In addition, seasonal AR(P) and seasonal MA(Q) terms account for correlations with seasonal lags. For this study, seasonality was analyzed over a time frame of months, i.e., there were 12 time periods in one season(Seasonality for the model is 12 months). The terms’ D’ and ‘d’ indicate the number of seasonal differencing and regular differencing, respectively. They were used to make the mean of the data series stationary. The term ‘s’ indicates the seasonality.

The SARIMA model specification is

$$\left(1-\phi_{1}B\right)\left(1-\Phi_{1}B^{12}\right)\left(1-B\right)\left(1-B^{12}\right)y_{t}=\left(1+\theta_{1}B\right)\left(1+\beta_{1}B^{12}\right)e_{t}$$

Where (1 − *ϕ*_1_*B*) = non-seasonal AR(1), (1 − *ϕ*_1_*B*^12^) = seasonal AR(1), (1 − B) = non-seasonal difference, (1 − B^12^) = seasonal difference, y_t_ = forecasted value, (1 + *θ*_1_*B*) = non-seasonal MA(1), (1 + *β*_1_*B*^12^) = seasonal MA(1), e_t_ = error term.

### Multivariate analysis

The autoregressive distributed lag (ARDL) was selected as the method to create a multivariate model. ARDL technique can be used for variables with a mixed order of integration. Irrespective of the difference order—I(0) or I(1) or a combination—ARDL models can be applied. Also, all underlying variables stand as a single equation in ARDL models [29]. In the data set, the variables of rainy days and relative humidity achieved a stationary mean in the first difference{I(1)}. The rest of the three variables, including the dependent variable, were stationary at the level{I(0)} (Tables 1 & 4).

The ARDL (p,q_1_,q_2_,q_3_,q_4_) model specification is

$$\phi \left(L,p\right)y_{t}=\sum_{i=1}^{k}\beta_{i}(L,q_{i})x_{it}+\delta w_{t}+u_{t}$$

where

$$\phi \left(L,p\right)=1-\phi_{1}L-\phi_{2}L^{2}-\ldots -\phi_{p}L^{p};$$

and

$$\beta \left(L,q\right)=1-\beta_{1}L-\beta_{2}L^{2}-\ldots -\beta_{q}L^{q},for\;I=1,2,3,\ldots ..,k,ut\sim iid(0;\delta^{2})$$

where p = number of patients; q_1_, q_2_, q_3_, and q_4_ represent rainfall, rainy days, relative humidity, and temperature, respectively; L = the lag operator and w_t_ = the vector of deterministic variables.

### Model validation

Serial autocorrelation of the SARIMA model was detected using Durbin Watson (DW) Statistic. DW around 2 is considered as no autocorrelation. To identify serial autocorrelation of the best-**[**fitting**]**multivariate model, the Lagrange multiplier (LM) test was applied. The LM test is the product of the coefficient of determination (R^2^) from auxiliary regression and the sample size, LM = nR^2^, where R^2^ = coefficient of determination and n = number of time points. A non-significant (i.e., *P***>** 0.05) value for the LM test indicates that the residuals of the time series model are not serially autocorrelated, and the Jarque-Bera(JB) test was used to detect the normality of residuals. The ARCH test was used to determine the heteroskedasticity of the residuals. In addition, the lowest Akaike information criterion (AIC) and the lowest mean absolute percentage error (MAPE) were used to select the best-fitting models. The formulas for calculating JB statistic, MAPE, and AIC are as follows;

$$JB\;test\;statistic=n\left[\frac{S^{2}}{6}+\frac{{\left(K-3\right)}^{2}}{24}\right]$$

where S = skewness and K = kurtosis.

$$MAPE=\frac{1}{n}\sum_{t=1}^{n}\left|\frac{O_{t-}y_{t}}{O_{t}}\right|$$

where n = number of time points, O_t_ = observed value, y_t_ = forecasted value

$$\text{AIC}=-\left(\text{log-likelihood}\right)+2K$$

where **K** is the number of model parameters.

$$DW=\frac{\sum_{t=2}^{T}{\left(e_{t}-e_{t-1}\right)}^{2}}{\sum_{t=1}^{T}e_{t}^{2}}$$

where e_t_ is the residual figure, and T is the number of observations in the experiment.

### Ethics statement

This study includes only secondary data. There is no significant ethical issue. Therefore, ethical clearance was not obtained for this study.

## Results

### Univariate analysis

**Fig 3:**
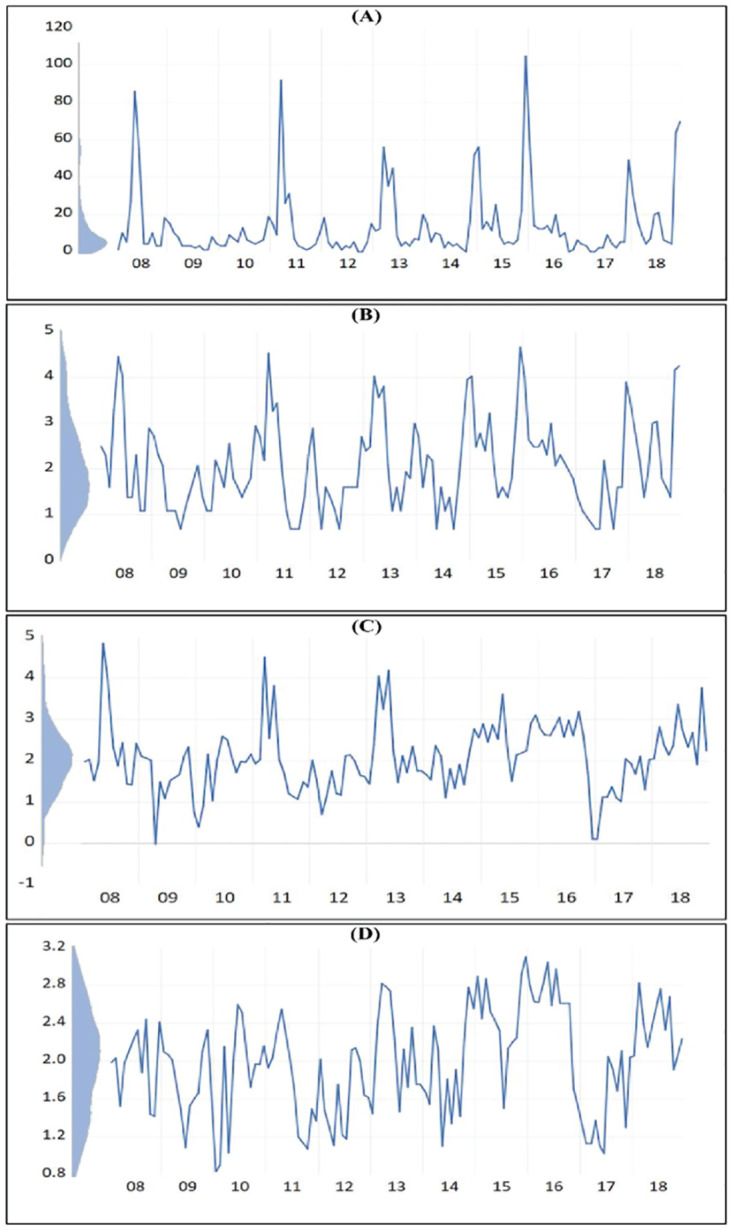
A-D: Distribution of monthly leptospirosis cases from January 2008 to December 2018 in Anuradhapura District, Sri Lanka, with kernel density. (A-Original distribution; B-Natural-log transformed; C-natural-log transformed and seasonally adjusted; D-natural-log transformed, seasonally adjusted and seasonal outliers adjusted).

Monthly leptospirosis cases in the Anuradhapura district from January 2007 to April 2019 are displayed in Fig 3A. Seasonally is a predominant feature in the distribution. Kernel density indicates that most of the values are within a certain limit, and therefore, the distribution peaks can be considered as positive outliers. However, these outliers were observed neither seasonally nor in regular intervals.

As the variance of the original distribution is not stationary, the variable is transformed into natural logarithm to achieve the variance stationarity and the results are displayed in Fig 3B [30]. Also, seasonality is more evident than in the original distribution after transformation. Nevertheless, the outliers of the distribution were still obvious even after transformation but were less prominent than the original distribution. Next, the Autocorrelation function (ACF) and the partial autocorrelation function (PACF) were plotted to confirm the seasonality. In addition, a seasonal unit root test was performed. The autocorrelation function and the partial autocorrelation function of the log-transformed variable are shown in S1 File. The first two lags were clearly significant in both ACF and PACF. In addition, lag 14–18, lag 24–25, and 34 to 36 were noticeably significant in ACF, indicating the seasonality of the distribution. Further, we applied the unit root tests to the original variable to confirm the stationarity of the mean and the seasonality. Table 1 shows the unit root test results for stationary mean and seasonality. According to the unit root test for the stationary mean, the mean is stationary in the series. At the same time, seasonality is significant in the series at a 5% significant level but not at a 1% significance.

As seasonality was significant in the variable, seasonal differencing was performed on the log-transformed variable. Log-transformed, seasonally differenced series was displayed in Fig 3C. Visually, seasonality is less evident in Fig 3C than in Fig 3B. However, outliers have become more prominent in seasonality adjusted series. The kernel density of Fig 3C indicates that more values are scattered at the vertical center while extending vertically towards the outliers. Therefore, the outliers were adjusted and interpolated using the linear function of Eviews software as described in the methodology section, and the adjusted series is displayed in Fig 3D. The kernel density of 3D indicates that the values are distributed evenly in the vertical axis compared to Fig 3C. To confirm that the seasonal differencing was successful, we repeated the seasonal unit root test for the seasonally differenced series. The results are displayed in S2 File. According to this, the test statistic increased from 9.8 to 15.4. Thus, the previous gap between the 5% significant level and the test statistic has been reduced. This indicates that the seasonal differencing has an effect on the variable. However, seasonality is still not significant at 1% significant level.

**Fig 4:**
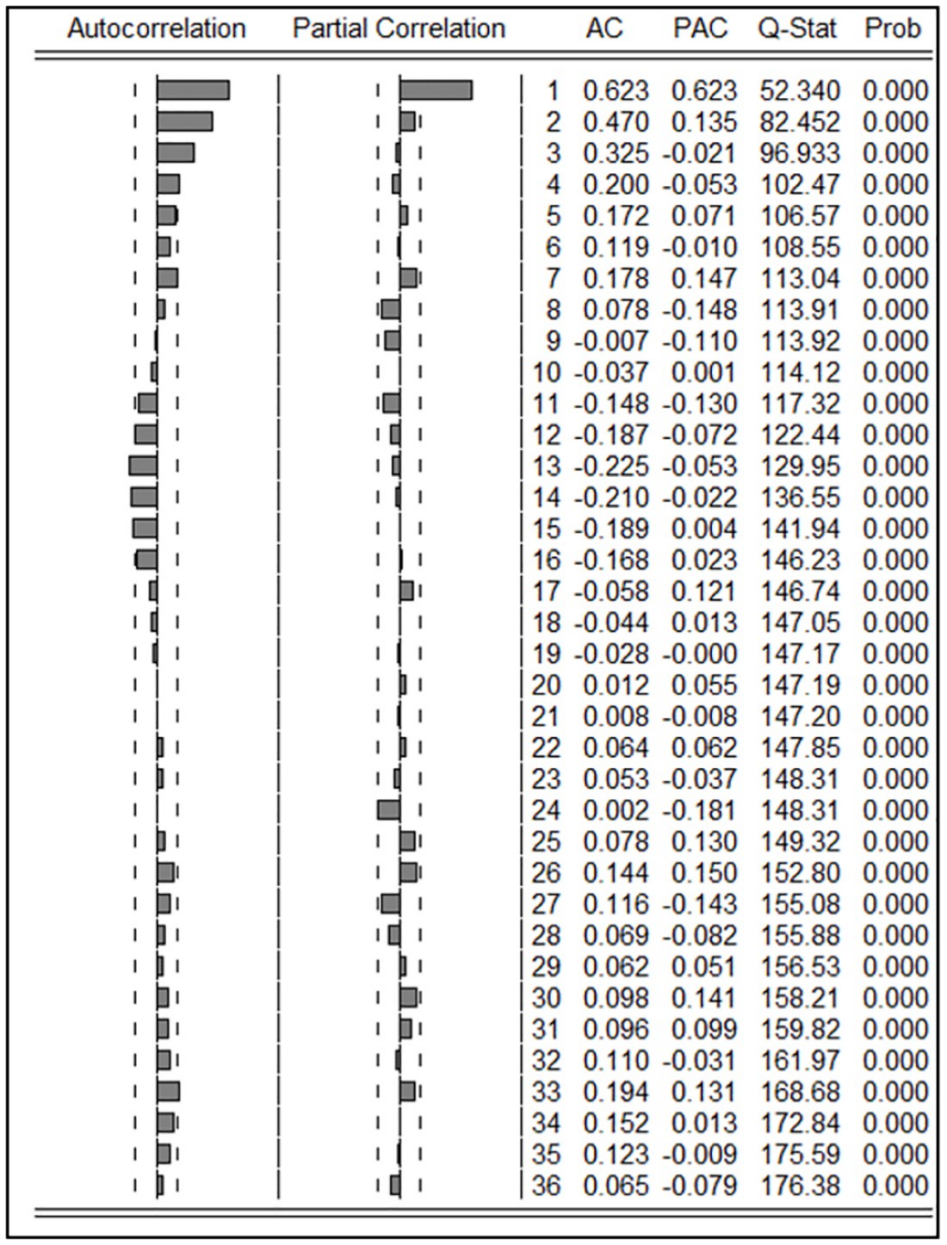
Autocorrelation function and partial autocorrelation function of seasonality adjusted; natural log-transformed monthly leptospirosis cases from January 2008 to December 2018, up to 36 lags. AC-Autocorrelation, PAC-Partial autocorrelation, Q-Stat-Ljung-Box test statistic, Prob-Probability (P-value).

The ACF and PACF of log-transformed, seasonally differenced and, outlier adjusted series were plotted and displayed in Fig 4. The first lag in PACF and four lags in ACF were significant. However, significant first lag in PACF and exponential decay in ACF indicate AR(1) process. Also, MA(4) is also a distant possibility according to the correlogram. Significant lag 12 of ACF indicates a MA(12) or SMA(1) process. In PACF, lag 12 is not significant, while lag 24 is significant, indicating doubtful results regarding the seasonal AR process. Considering all these possibilities, the following ARMA models were fitted for the data series, and the results are displayed in Table 2.

**Table 2:** Comparison of univariate seasonal ARIMA models for the monthly leptospirosis cases of Anuradhapura District, Sri Lanka.

Model	Log-likelihood	AIC	BIC	HQ
**ARIMA(1,0,0)(0,1,1)**_**12**_	**-35.1**	**1.08**	**1.21**	**1.13**
ARIMA(1,0,0)(2,1,1)_12_	-34.7	1.13	1.32	1.20
ARIMA(1,0,0)(1,1,1)_12_	-35.1	1.11	1.27	1.17
ARIMA(1,0,0)(2,1,2)_12_	-34.7	1.16	1.38	1.24
ARIMA(1,0,4)(0,1,1)_12_	-33.4	1.15	1.40	1.25
ARIMA(1,0,4)(0,1,2)_12_	-33.4	1.17	1.46	1.29
ARIMA(1,0,4)(1,1,2)_12_	-33.1	1.19	1.51	1.32
ARIMA(1,0,4)(2,1,2)_12_	-33.4	1.23	1.58	1.37

AIC- Akaike information criterion, BIC- Bayesian information criteria, HQ-Hannan-Quinn information Criteria

According to Table 2, the best model to predict monthly leptospirosis cases in the Anuradhapura district was ARIMA(1,0,0)(0,1,1)_12._ Seasonality, AR(1) and SMA(1) have been detected as the prominent features through plotting ACF and PACF. In addition, this model has the lowest values of **AIC (**Akaike information criterion**), BIC** (Bayesian information criteria)**, and HQ (**Hannan-Quinn information). Further details on the best-fitted model are displayed in Table 3. Durbin Watson test statistic was 1.97, which was closer to, two and it indicates that there is no residual serial correlation. The ARCH test result was not significant, indicating heteroskedasticity was not observed in the residuals after fitting the model. (Table 3) Non-significant results of the Jarque-Bera test indicated that the residual follows a normal distribution. (S3 File) In addition, ACF and PACF were plotted for the residuals of the best-fitted models. (S4 File) As none of the lags were significant in residual correlograms, we can predict that the model has adequately described the observed variability. All other models displayed in Table 3 also described the observed variability satisfactorily; however, ARIMA(1,0,0)(0,1,1)_12_ is the parsimonious model with a lesser number of parameters. The model specification of the best fitted model [ARIMA(1,0,0)(0,1,1)_12_] was;

$$\left(1-\phi_{1}B\right)\left(1-B^{12}\right)y_{t}=\left(1+\beta_{1}B^{12}\right)e_{t}.$$

**Table 3:** Model description of best fitted, univariate seasonal ARIMA model for the monthly leptospirosis cases of Anuradhapura District, Sri Lanka.

Model	Parameter	Coefficient	Model Statistics	Model Validation
ARIMA(1,0,0)(011)_12_ Model fitting 2008Jan– 2013Dec Model validation 2014Jan– 2018Dec	C	1.82	Ad. R2–0.27 F– 10.1 P– 0.0001	MAPE– 19.8 Serial Correlation Durbin Watson– 1.97 Residual Normality Jarque-Bera– 1.27 P– 0.52 Residual Heteroskedasticity(ARCH test (12 lags) F—1.43 P– 0.18
	AR	0.37		
	SMA(1) (MA(12))	-0.37		
	Seasonal factor	1		

**Fig 5:**
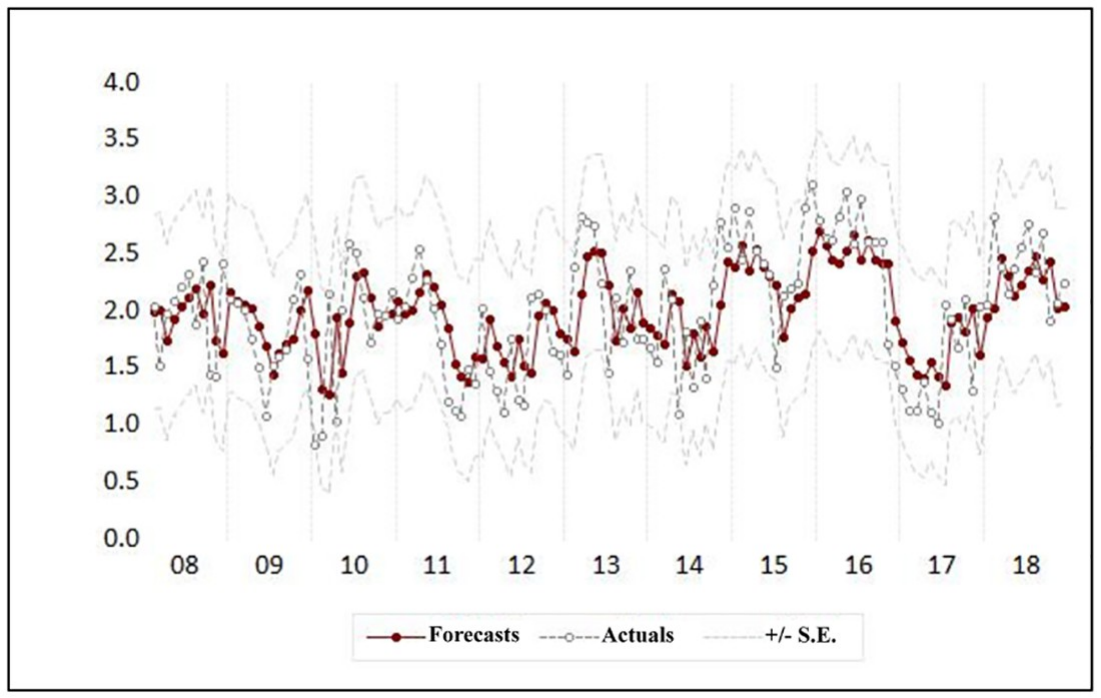
Comparison of forecasted and actual leptospirosis cases by univariate seasonal ARIMA model.

Fig 5 shows the forecasted values and the observed monthly leptospirosis cases. However, MAPE value of the model was 19.8. (Table 3).

### Multivariate analysis

**Fig 6:**
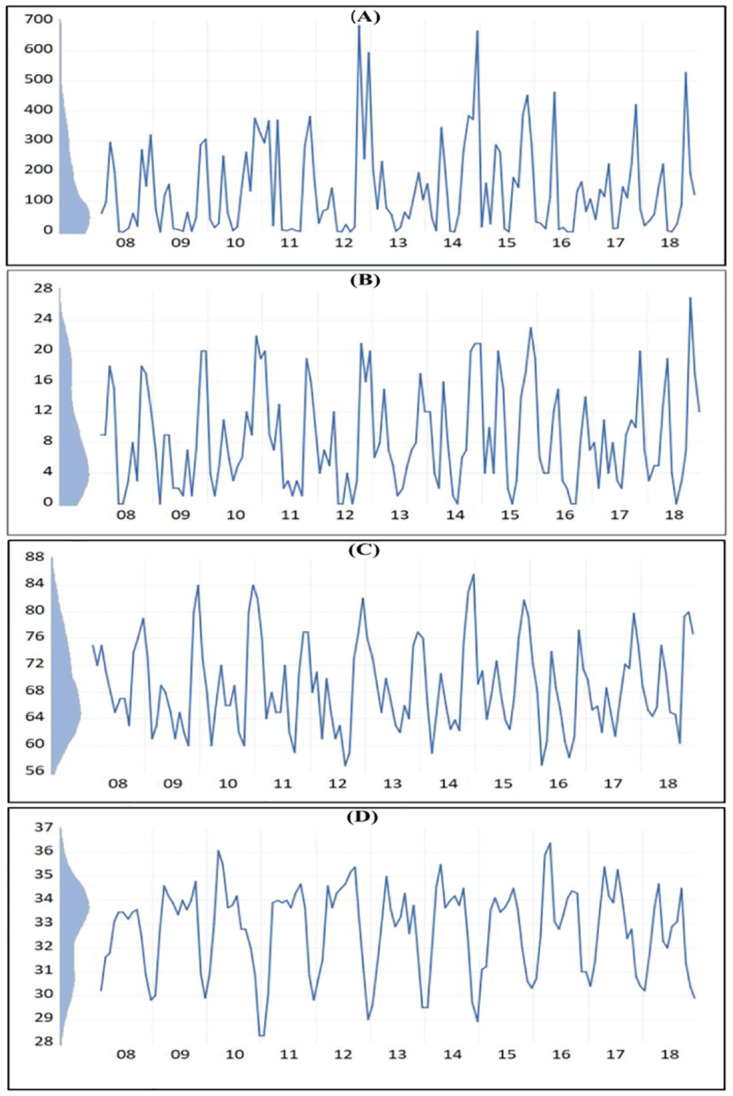
A-D: Distribution of monthly meteorological parameters from January 2008 to December 2018 in Anuradhapura District, Sri Lanka, with kernel density. (A-Rainfall; B-Rainy days; C-day time average relative humidity; D-maximum temperature).

Fig 6A–6D show the distribution of monthly rainfall, rainy days, average daytime relative humidity, and the maximum temperature of the Anuradhapura district from January 2008 to December 2018.

The results of the unit root tests of meteorological variables are displayed in Table 4. Accordingly, Rainfall and Temperature were stationary without differencing, while rainy days and relative humidity were stationary at the first difference. Therefore, including the dependent variable, three variables were stationary at I(0), and two variables were stationary at I(1).

**Table 4:** Unit root test results of the independent variables for the multivariate analysis.

Variable	Unit root statistic (Level)	P-value	Unit root statistic (1^st^ Difference)	P-value
Rainfall	-8.7	<0.000		
Rainy days	-2.4	0.12	-10.4	<0.0001
Relative Humidity	-2.7	0.06	-10.4	<0.0001
Temperature	-2.9	0.04	-12.2	<0.0001

The lag length of criteria to determine the best predictive lag length was observed for each independent variable separately and as a whole (S4 and S5 Files). The third lag was considered the best lag for plotting the ARDL model.

ARDL models were fitted with a lag length of three, and the best selected ARDL models were displayed in Table 5. Table 6 shows the criteria of the best-selected model. ARDL(1,3,2,0,1) was selected as the best model to describe the observed variability with the lowest AIC value.

**Table 5:** Best fitted ARDL models with minimum AIC values.

Model	Log-likelihood	Adjusted R^2^	AIC
ARDL(1, 3, 2, 0, 1)	-58.5	0.57	2.044
ARDL(1, 3, 2, 1, 3)	-55.8	0.58	2.054
ARDL(1, 3, 2, 3, 0)	-56.9	0.58	2.054
ARDL(1, 0, 2, 2, 3)	-58.1	0.57	2.061
ARDL(1, 0, 2, 1, 3)	-59.1	0.57	2.061
ARDL(1, 0, 3, 1, 0)	-61.1	0.56	2.062
ARDL(1, 3, 2, 2, 0)	-58.2	0.57	2.064

**Table 6:** Model description of the best model [ARDL(1,3,2,0,1)].

Variable	Lag	Coefficient	T statistic	P-value	Model validation
**Patients**	-1	0.28	2.77	0.0074	Adjusted R^2^–0.57 F statistic– 9.35 P– 0.0000 AIC– 2.04 SC– 2.43 HQC– 2.19 Residual Normality Jarque-Bera– 2.60 P—0.27 Residual Heteroskedasticity(ARCH) F– 1.13 P—0.35 Residual Serial correlation(LM test– 2 lags) F– 0.02 P– 1.00 MAPE– 30.8
**Rainfall**	0	-0.22	-1.14	0.2556	
**Rainfall**	-1	-0.02	-0.14	0.8830	
**Rainfall**	-2	0.31	1.88	0.0651	
**Rainfall**	-3	0.31	2.41	0.0189	
**Rainy days**	0	0.61	2.45	0.0172	
**Rainy days**	-1	0.57	2.50	0.0152	
**Rainy days**	-2	0.39	1.86	0.0676	
**Relative Humidity**	0	-0.50	-2.32	0.0234	
**Temperature**	0	0.11	0.56	0.5775	
**Temperature**	-1	0.39	2.34	0.0226	
**Constant**	-	1.40	6.30	<0.0000	

According to Table 6, the model has significantly described the observed variability. Patient numbers in the previous month significantly predicted leptospirosis in this month. Also, the average number of rainy days per month showed a positive association with patient numbers in lag 0, 1, and 2. However, only lag 2 and 3 of rainfall showed a positive association with leptospirosis. Relative humidity displayed a negative effect on leptospirosis. The temperature showed a positive association with the occurrence of leptospirosis.

Residuals were checked for normality, heteroskedasticity and serial correlation to validate the ARDL model, and the residuals follow a normal distribution. (S6 File) Furthermore, according to Table 6, the ARCH test and LM test were not significant, indicating that the residuals do not show heteroskedasticity and no serial correlation of the residuals. None of the lags were significant in the residual ACF and PACF, indicating the validity of the fitted model.

**Fig 7:**
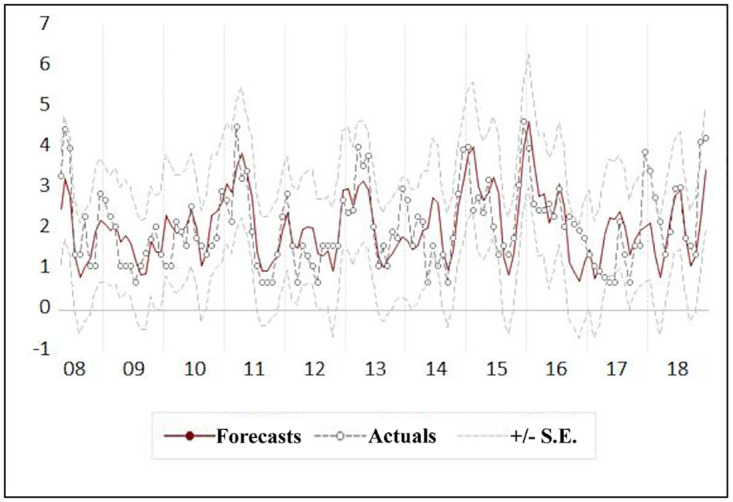
Comparison of forecasted and actual leptospirosis cases by multivariate seasonal ARIMA model.

Fig 7 shows the forecast values for the observed monthly leptospirosis cases by the ARDL model.

Table 7 depicts the results of the best-fitted models with and without seasonal or general outliers. The best-fitted models described above are in bold. The effect of outlier adjustment for a model is discussed in detail in the discussion section.

**Table 7:** Final model comparison.

Model Category	Outlier adjustment	Best fitted Model	MAPE	AIC value	Res: nor:P	Res: hete:P	Res: SC*
Univariate	General outliers	ARIMA(1,0,0)(0,1,0)_12_	23.1	1.57	0.00	0.78	DW^#^-2.00
	**Seasonal outliers**	**ARIMA(1,0,0)(0,1,1)**_**12**_	**19.8**	**1.08**	**0.52**	**0.18**	**DW** ^#^ **-1.97**
	No outliers	ARIMA(1,0,0)(1,1,1)_12_	28.82	2.39	0.00	0.46	DW^#^-2.00
Multivariate	General outliers	ARDL(1, 3, 1, 0, 1)	26.1	1.54	0.60	0.45	LM^#^-0.17
	Seasonal outliers	ARDL(1, 3, 1, 3, 0)	17.9	1.14	0.44	0.36	LM^#^-0.82
	**No** outliers	**ARDL(1, 3, 2, 0, 1)**	**30.8**	**2.04**	**0.27**	**0.35**	**LM** ^#^ **-1.00**

Res-Residual; P-P value; SC-serial correlation; nor-normality; here-heteroskedasticity; DW-Durbin-Watson test;

*To assess the serial correlation of univariate models Durbin-Watson test was used, and for multivariate models, LM test was used;

^#^value of DW statistic and a P-value of LM test

## Discussion

Using the Autoregressive distributed lag (ARDL) method, we showed that the leptospirosis case could be predicted using metrological parameters and the previous month case numbers in the dry zones in Sri Lanka. ARDL, VAR, VECM, ARCH, and GARCH are the possible models that can be used to determine the association of these meteorological parameters and to predict leptospirosis. In this analysis, the Autoregressive distributed lag(ARDL) method was selected over other techniques based on the mean stationarity of the five variables. However, ARDL models are not often used for disease predictions. We have shown that the ARDL models can be used for disease predictions as naturally existing variables cannot be expected to be in the same order of integration.

As Abraham *et al*. [31] proposed, we performed methodological changes in this model by adjusting the outliers after seasonal differencing instead of performing from the beginning. These outliers were observed neither seasonally nor in regular intervals (Fig 3C). Therefore, these outliers observed during the non-leptospirosis season of the time series can directly influence the lack of fitting of the model. Univariate models cannot capture the influence of these external factors and multivariate models are needed to explain the influence of external factors on the occurrence of leptospirosis.

The observed MAPE values are slightly higher than the accepted range in both univariate and multivariate models. This could be due to the non-inclusion of non-meteorological regressors such as human behavior-related factors into the model. As described in Table 7, residual heteroskedasticity and residual serial correlation were not observed in any models. However, the residual normality could only be achieved after seasonal outlier adjustments in the univariate models, while the residuals were normally distributed in multivariate models. As shown in Table 7, the best-fitted model was observed in the seasonality-adjusted series. (Lowest MAPE and AIC values) and a valid model could only be fitted to original data (without outlier adjustments) by multivariate modelling. It was evident that the seasonal outliers are a major reason for the mal-fitting of the models containing seasonal data. Leptospirosis outbreaks generally occur during the rainy season, extending from November to January. Thus, any outbreak during the dry season could be identified as a seasonal outlier. Therefore, adjusting the outliers in the original variable is not the best option in a seasonal data series. Higher case numbers occur during a rainy season may not be an outlier, while even a small peak in the non-rainy season could be an outlier and cause the mal-fitting of the model. The techniques and their order used for outlier adjustments were not discussed adequately in previous publications.

The average number of rainy days per month was associated with patient numbers in lag 0, 1, and 2. However, only lag 2 and 3 of rainfall was associated with leptospirosis. The standardized coefficients are also higher in rainy days than in rainfall. This might be due to continuous rain over a period of a few months, creating a favourable environment for the growth and survival of *Leptospira*. This finding is consistent with our previous work [5]. However, many previous studies suggest that rainfall is vital in predicting leptospirosis [15, 32, 33]. Most of these studies have not adjusted the collinearity effect of rainfall and rainy days. A study conducted in China showed that limited rainfall is associated with leptospirosis while heavy rainfall is not associated, probably because heavy rainfall can be negatively associated with leptospirosis as the natural habitats of rodents are destroyed [34].

In contrast to the previses published work which showed a lack of association between relative humidity and leptospirosis [5], our prediction model shows a negative association. Several studies suggest that warm, humid conditions are needed to survive *L*. *interrogans* outside the host [16, 35]. However, some studies suggest that a higher relative humidity has a negative impact on rodents which is the natural host of *Leptospira* [36]. Also, evidence suggests that infective *Leptospira* species in different geographical regions could be different and some of the essential requirement for survival is different [21]. These facts may partially explain the observed association. The temperature shows a positive association with the occurrence of leptospirosis. Although this is a contrasting finding compared to previous studies, some suggest that temperature is associated with rodents’ survival and rodent densities [35].

As mentioned in the introduction section, most of the time series models were univariate and developed targeting the districts in the wet zone of Sri Lanka. In comparison to the wet zone, accuracy of the univariate models seems less in the dry zone. As mentioned above, this could be attributed to the other factors influencing leptospirosis in the dry zone, while in the wet zone, leptospirosis mostly depends on the meteorological factors [5]. The highest coefficient observed for the constant clearly indicates that there are other factors influencing leptospirosis occurrence. This indicates that at least a few other major parameters are required to create better models to predict leptospirosis [37]. Leptospirosis disease transmission is a complex and dynamic process that involves human behaviours, susceptibility, non-human host behaviours and reproduction, growth and survival of *Leptospira* in the natural environment and the ecology of the concerning geography. All these factors could not be fitted to a single model. However, as mentioned above, these parameters could be included in the models developed in future studies. Despite the limitations mentioned, these models could effectively predict leptospirosis not only in the Anuradhapura district but also in the other districts of the dry zone with similar meteorological characteristics. In addition, these models could be validated further in other countries which share similar meteorological and sociodemographic characteristics.

In conclusion, we have demonstrated that seasonal outliers are a significant limitation of creating univariate models in relatively dry environments. In contrast, multivariate models had captured the seasonal outliers and showed residual normality, indicating that all the outliers were due to the effect of meteorological parameters. Leptospirosis cases in the previous month, rainfall, rainy days, and temperature had shown a positive association with leptospirosis, while relative humidity indicated a negative association. These models could be effectively utilized in public health policy and planning using the routinely available data. We propose further studies incorporating other contributory factors in addition to meteorological variables for better disease prediction.

## Supporting information

S1 File Autocorrelation function and partial autocorrelation function of natural log-transformed monthly leptospirosis cases from January 2008 to December 2018 up to 36 lags. (DOCX)

S2 File Standard and seasonal unit root test results of seasonality adjusted; natural log-transformed monthly leptospirosis cases. (DOCX)

S3 File Frequency distribution of residuals of the univariate model (residuals follow the normal distribution, P = 0.52). (DOCX)

S4 File Lag length criteria to determine the best ARDL model for the individual variables. (DOCX)

S5 File Lag length criteria to determine the best ARDL model. (DOCX)

S6 File Frequency distribution of residuals of the univariate model (residuals follow the normal distribution, P = 0.27). (DOCX)

## Abbreviations

ACF: Autocorrelation function
AD: Anderson Darling test
ADF: AugmentedDickey-Fuller test
AIC: Akaike information criterion
AR: Auto-Regressive
ARCH: Auto-Regressive conditional heteroscedasticity
ARDL: Auto-Regressive distributed lag
ARIMA: Auto-Regressive integrated moving average
ARIMAX: Multivariate autoregressive integrated moving average with input series
ARMA: Auto-Regressive moving average
BIC: Bayesian information criteria
DALY: Disability-adjusted life years
DW: Durbin Watson test
GARCH: Generalised Auto-Regressive conditional heteroscedasticity
HEGY: Hylleberg, Engle, Granger, and Yoo
HQ: Hannan-Quinn information
IMMR: Indoor Mortality and Morbidity Report
IQR: Inter quartile range
JB: Jarque-Bera test
LM: Lagrange multiplier
MA: Moving Average
MAPE: Mean absolute percentage error
PACF: Partial autocorrelation function
PGIA: Postgraduate Institute of Agriculture
Qstat: Ljung-Box test statistic
RD: Rainy days
RF: Rainfall
RH: Relative humidity
RMSE: Root mean square error
SARIMA: Seasonal Autoregressive integrated moving average with input series
SBC: Schwartz’s Bayesian Criterion
SMA: SeasonalMoving Average
SPSS: Statistical package for social sciences
TEM: Temperature
UV: Ultraviolet
VAR: Vector Auto-Regressive
VECM: Vector error correction model
VIF: Variance inflation factor
