# Supplementary material for: SARIMA and ARDL models for predicting leptospirosis in Anuradhapura district Sri Lanka
Source: PLoS One. 2022 Oct 13;17(10):e0275447. doi: 10.1371/journal.pone.0275447 (PMC9562162; doi:10.1371/journal.pone.0275447)
Supplement: S1 File — (DOCX) [file pone.0275447.s001.docx]

**Supplementary file 1**


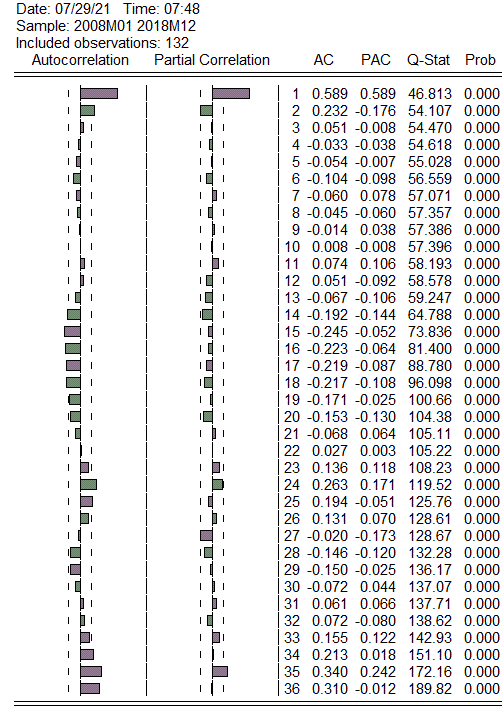


*AC-Autocorrelation, PAC-Partial autocorrelation, Q-Stat-Ljung-Box test statistic, Prob-P value)*

**Autocorrelation function and partial autocorrelation function of natural log-transformed monthly leptospirosis cases from January 2008 to December 2018 up to 36 lags.**

**Supplementary file 2**

**Standard and seasonal Unit root test results of seasonality adjusted; natural log-transformed monthly leptospirosis cases.**

|  | **Traditional HEGY process for stationary seasons** |
| --- | --- |
| **Frequency** | **12 months per cycle** |
| **1% significance level** | 33.9 |
| **5% significance level** | 8.15 |
| **Test statistic** | 15.4 |
| **Interpretation** | Seasonality is still significance at 1% significance |

**Supplementary file 3**

**
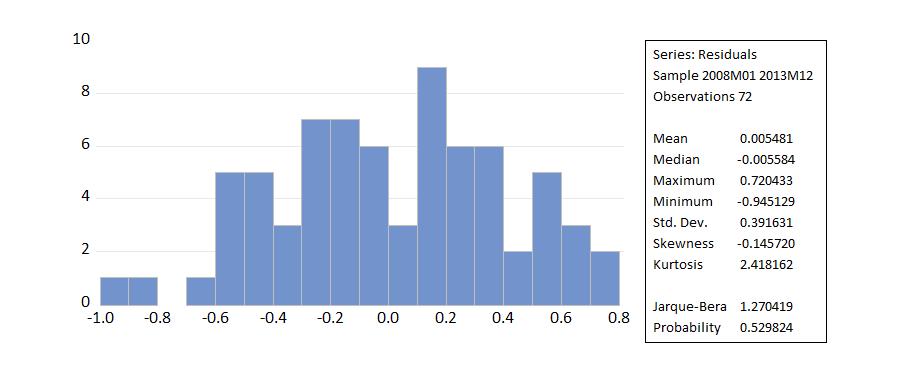
**

**Frequency distribution of residuals of the Univariate Model (Residuals follow the normal distribution, P=0.52)**

**Supplementary file 4**

**Lag length criteria to determine the best ARDL model for the individual variables**

| **Variable** | **Best Lag** | **Log-likelihood** | **AIC** |
| --- | --- | --- | --- |
| **Leptospirosis patients** | **2** | **-138.7** | **2.28** |
| **Rainfall** | **2** | **-280.0** | **4.83** |
| **Rainy days** | **3** | **-261.8** | **4.59** |
| **Relative Humidity** | **2** | **-257.3** | **4.45** |
| **Temperature** | **2** | **-234.6** | **4.14** |

**Supplementary file 5**

**Lag length criteria to determine the best ARDL model**

| **Lag** | **Log-likelihood** | **Final Prediction Error** | **AIC** |
| --- | --- | --- | --- |
| 0 | -603.3 | 0.0174 | 10.14 |
| 1 | -449.6 | 0.0020 | 7.99 |
| 2 | -399.6 | 0.0013 | 7.58 |
| 3 | -365.2 | 0.0012* | 7.42* |
| 4 | -352.7 | 0.0014 | 7.63 |
| 5 | -329.0 | 0.0015 | 7.65 |
| 6 | -313.0 | 0.0018 | 7.80 |
| 7 | -292.4 | 0.0020 | 7.87 |
| 8 | -270.8 | 0.0022 | 7.93 |
| 9 | -252.6 | 0.0026 | 8.04 |
| 10 | -229.8 | 0.0030 | 8.08 |
| 11 | -206.2 | 0.0034 | 8.10 |
| 12 | -181.6 | 0.0039 | 8.11 |

***(All meteorological parameters are the regressors, third lag was the best lag with lowest AIC)***

**Supplementary file 6**

**
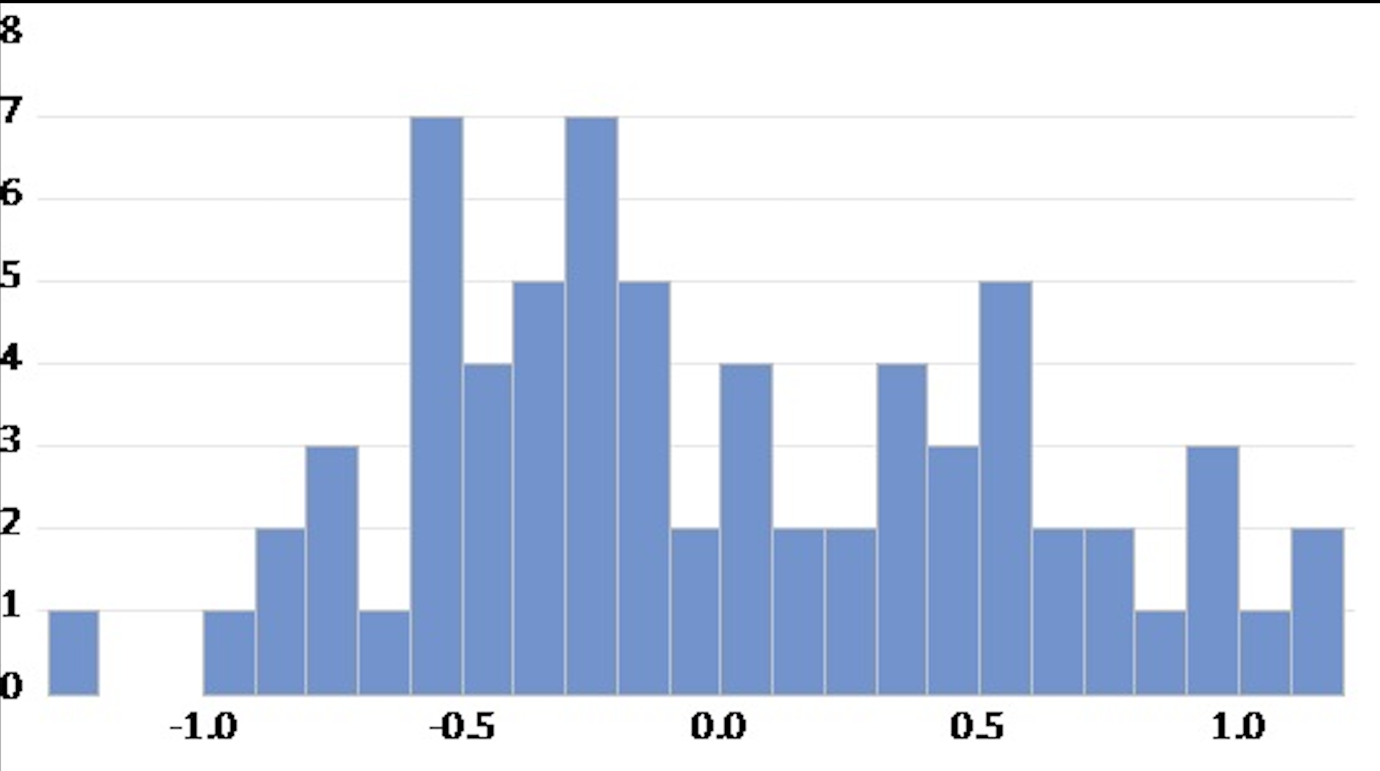
**

**Frequency distribution of residuals of the Univariate Model (Residuals follow the normal distribution, P=0.27)**
