# Supplementary material for: SARIMA and ARDL models for predicting leptospirosis in Anuradhapura district Sri Lanka
Source: PLoS One. 2022 Oct 13;17(10):e0275447. doi: 10.1371/journal.pone.0275447 (PMC9562162; doi:10.1371/journal.pone.0275447)
Supplement: S2 File — (DOCX) [file pone.0275447.s002.docx]

**Supplementary file 2**

**Standard and seasonal Unit root test results of seasonality adjusted; natural log-transformed monthly leptospirosis cases.**

|  | **Traditional HEGY process for stationary seasons** |
| --- | --- |
| **Frequency** | **12 months per cycle** |
| **1% significance level** | 33.9 |
| **5% significance level** | 8.15 |
| **Test statistic** | 15.4 |
| **Interpretation** | Seasonality is still significance at 1% significance |
