# Supplementary material for: SARIMA and ARDL models for predicting leptospirosis in Anuradhapura district Sri Lanka
Source: PLoS One. 2022 Oct 13;17(10):e0275447. doi: 10.1371/journal.pone.0275447 (PMC9562162; doi:10.1371/journal.pone.0275447)
Supplement: S3 File — (DOCX) [file pone.0275447.s003.docx]

**Supplementary file 3**

**
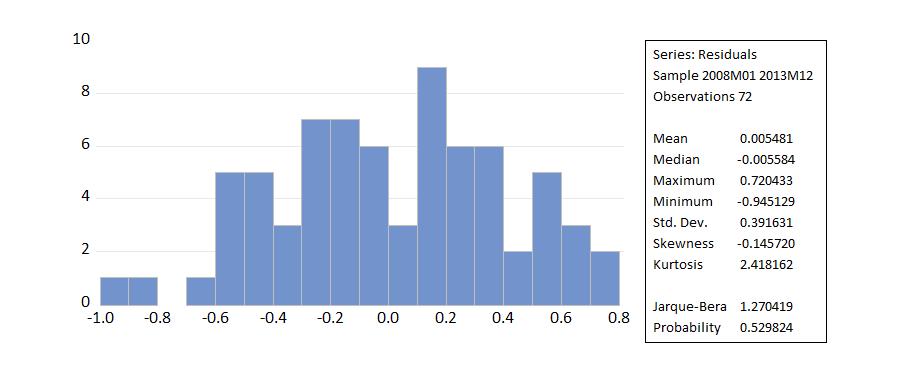
**

**Frequency distribution of residuals of the Univariate Model (Residuals follow the normal distribution, P=0.52)**
