# Supplementary material for: SARIMA and ARDL models for predicting leptospirosis in Anuradhapura district Sri Lanka
Source: PLoS One. 2022 Oct 13;17(10):e0275447. doi: 10.1371/journal.pone.0275447 (PMC9562162; doi:10.1371/journal.pone.0275447)
Supplement: S4 File — (DOCX) [file pone.0275447.s004.docx]

**Supplementary file 4**

**Lag length criteria to determine the best ARDL model for the individual variables**

| **Variable** | **Best Lag** | **Log-likelihood** | **AIC** |
| --- | --- | --- | --- |
| **Leptospirosis patients** | **2** | **-138.7** | **2.28** |
| **Rainfall** | **2** | **-280.0** | **4.83** |
| **Rainy days** | **3** | **-261.8** | **4.59** |
| **Relative Humidity** | **2** | **-257.3** | **4.45** |
| **Temperature** | **2** | **-234.6** | **4.14** |
