# Supplementary material for: SARIMA and ARDL models for predicting leptospirosis in Anuradhapura district Sri Lanka
Source: PLoS One. 2022 Oct 13;17(10):e0275447. doi: 10.1371/journal.pone.0275447 (PMC9562162; doi:10.1371/journal.pone.0275447)
Supplement: S5 File — (DOCX) [file pone.0275447.s005.docx]

**Supplementary file 5**

**Lag length criteria to determine the best ARDL model**

| **Lag** | **Log-likelihood** | **Final Prediction Error** | **AIC** |
| --- | --- | --- | --- |
| 0 | -603.3 | 0.0174 | 10.14 |
| 1 | -449.6 | 0.0020 | 7.99 |
| 2 | -399.6 | 0.0013 | 7.58 |
| 3 | -365.2 | 0.0012* | 7.42* |
| 4 | -352.7 | 0.0014 | 7.63 |
| 5 | -329.0 | 0.0015 | 7.65 |
| 6 | -313.0 | 0.0018 | 7.80 |
| 7 | -292.4 | 0.0020 | 7.87 |
| 8 | -270.8 | 0.0022 | 7.93 |
| 9 | -252.6 | 0.0026 | 8.04 |
| 10 | -229.8 | 0.0030 | 8.08 |
| 11 | -206.2 | 0.0034 | 8.10 |
| 12 | -181.6 | 0.0039 | 8.11 |

***(All meteorological parameters are the regressors, third lag was the best lag with lowest AIC)***
